# Supplementary material for: A framework for Frizzled-G protein coupling and implications to the PCP signaling pathways
Source: Cell Discov. 2024 Jan 5;10:3. doi: 10.1038/s41421-023-00627-y (PMC10770037; doi:10.1038/s41421-023-00627-y)
Supplement: Supplementary file 1 — Supplementary information, Figures and Tables [file 41421_2023_627_MOESM1_ESM.pdf]

Supplementary Figures

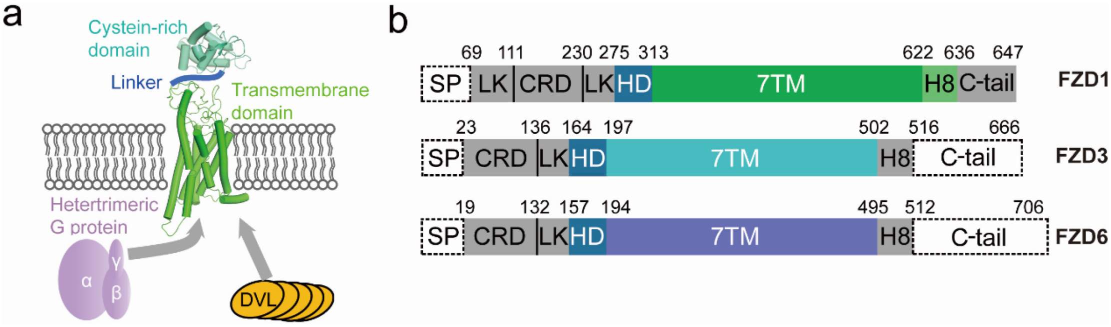

Supplementary Fig. S1 Schematic diagram of FZDs and schematic representation of FZD1, 3 and 6. a, Schematic diagram of the frizzled receptors (FZDs): a flexible linker domain (blue) connects the CRD (cyan) to the transmembrane domain (green). FZDs mainly transmit signals through Dishevelled (DVL) and possibly G proteins. **b,** Schematic representation of wild-type sequences of FZD1, 3 and 6. Numbers indicate amino acid numbering in the indicated FZD subtype. The colored region stands for residues observed in respective structures. SP: signal peptide; LK: linker; HD: hinge domain. The dashed lines indicate regions not included in the constructs; the grey zones indicate regions disordered in the structures.

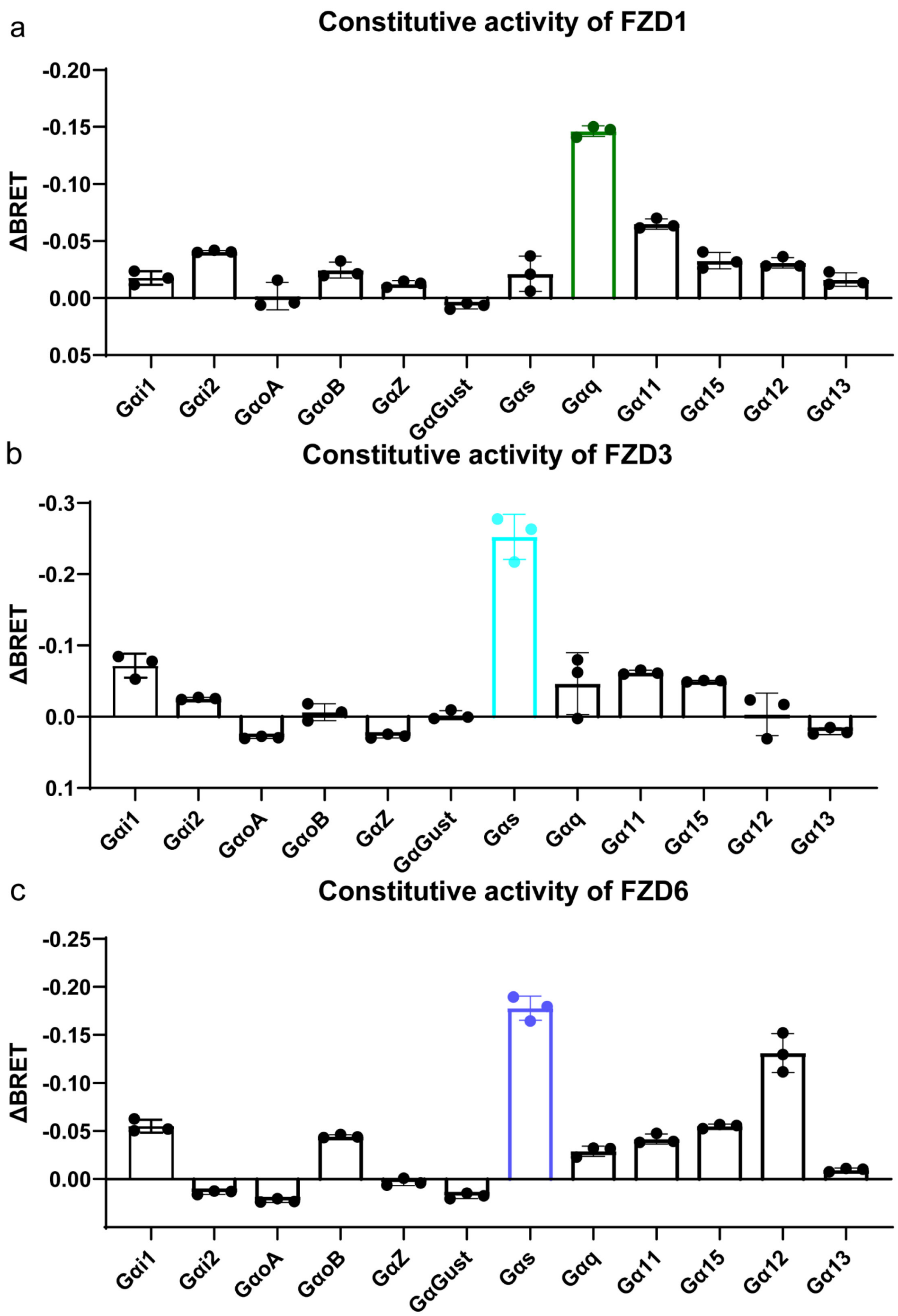

Supplementary Fig. S2 Constitutive activity of FZD1, FZD3 and FZD6 on different G proteins measured by BRET assay. The constitutive activity of FZD1 (**a**), FZD3 (**b**) and FZD6 (**c**) on different G proteins are measured by BRET assay. The G protein with the highest constitutive activity of each FZD was colored. ΔBRET represents the change of bioluminescence resonance energy transfer value: ΔBRET= BRET signal (GPCR-G protein sensor) − BRET signal (only G protein sensor). Data are mean ± s.e.m. (n = 3).

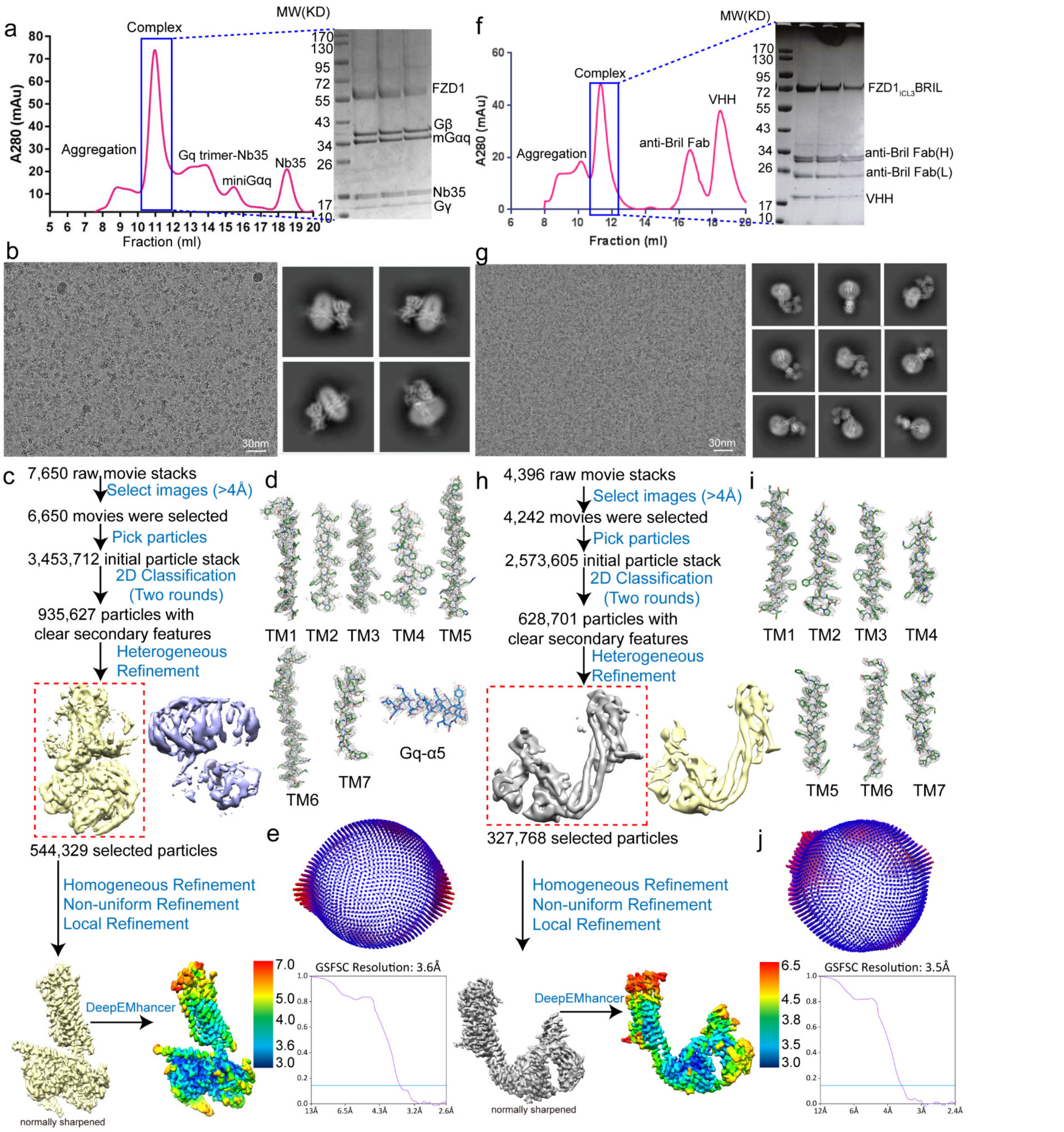

Supplementary Fig. S3 Cryo-EM sample preparation and data processing for FZD1 complexes. a, f, Elution profile and gel image of FZD1-Gq (**a**) and inactive FZD1 (**f**) complexes. **b, g,** Representative cryo-EM image and representative 2D averages of the FZD1-Gq (**b**) and inactive FZD1 (**g**) complexes. **c, h,** Cryo-EM data processing workflow of FZD1-Gq (**c**) and inactive FZD1 (**h**). The data was processed by CryoSPARC and final 3D density maps are colored according to the local resolution. Gold-standard FSC curves from CryoSPARC indicate overall nominal resolutions of 3.64 Å and 3.52 Å using the FSC = 0.143 criterion for the FZD1-Gq (**c**) and inactive FZD1 (**h**) structures. **d, i**, Cryo-EM density maps and models are shown for all transmembrane helices and α5 in the Gαq protein for FZD1-Gq (**d**) and inactive FZD1 (**i**) structures, respectively. **e, j**, Angular distribution of the particles used for the final reconstruction of the FZD1-Gq complex (**e**) and inactive FZD1 (**j**) complex.

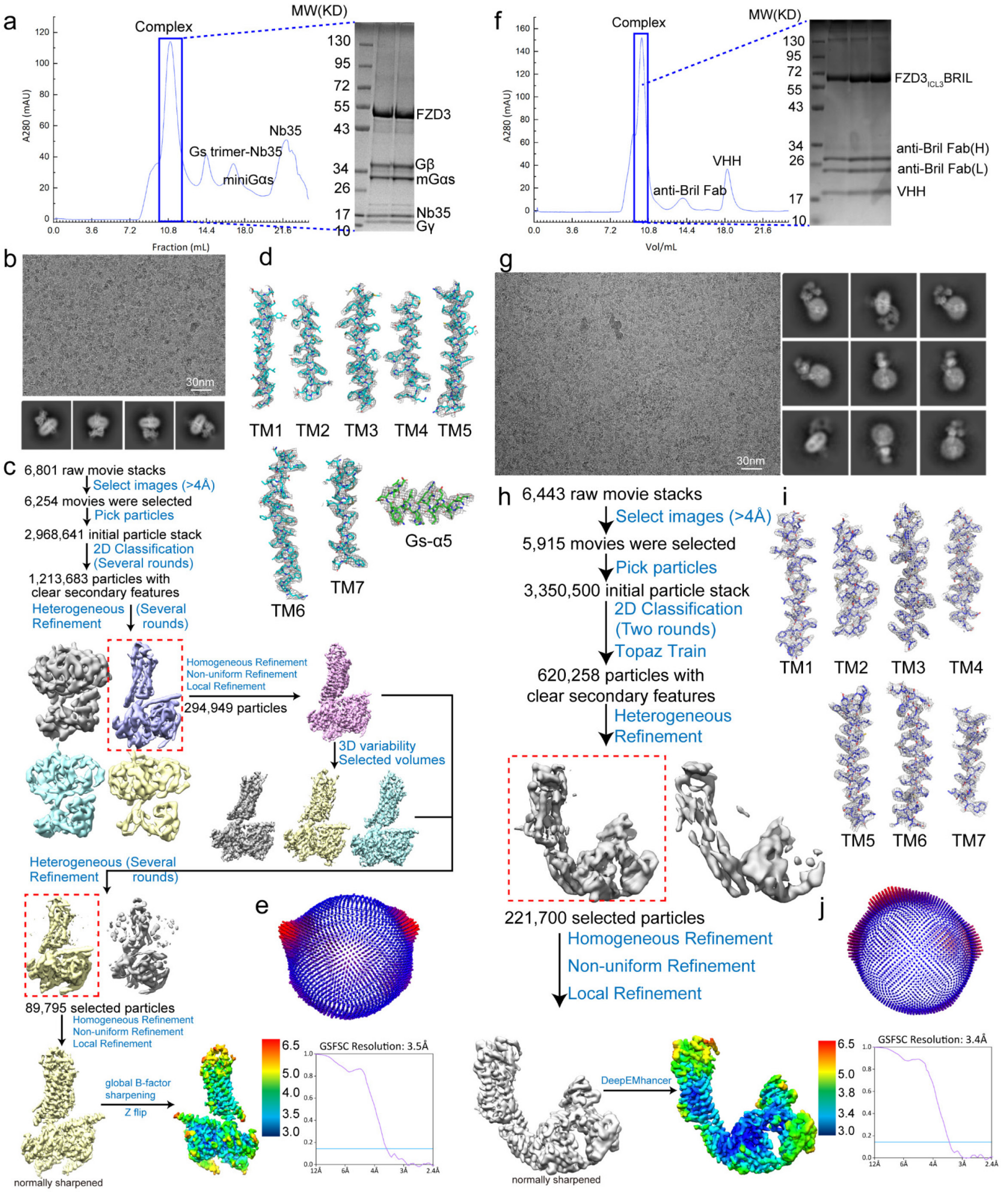

Supplementary Fig. S4 Cryo-EM sample preparation and data processing for FZD3 complexes. a, **f**, Elution profile and gel image of FZD3-Gs (**a**) and inactive FZD3(**f**) complexes. **b**, **g**, Representative cryo-EM image and representative 2D averages of the FZD3-Gs (**b**) and inactive FZD1 (**g**) complexes**. c**, **h**, Cryo-EM data processing workflow of FZD3-Gs (**c**) and inactive FZD3 (**h**). The data was processed by CryoSPARC and final 3D density maps are colored according to the local resolution. Gold-standard FSC curves from CryoSPARC indicate overall nominal resolutions of 3.50 Å and 3.38 Å using the FSC = 0.143 criterion for the FZD3-Gs (**c**) and inactive FZD3 (**h**) structures. **d**, **i**, Cryo-EM density maps and models are shown for all transmembrane helices and α5 in the Gαs protein for FZD3-Gs (**d**) and inactive FZD3 (**i**) structures, respectively. **e, j**, Angular distribution of the particles used for the final reconstruction of the FZD3-Gs complex (**e**) and inactive FZD3 (**j**) complex.

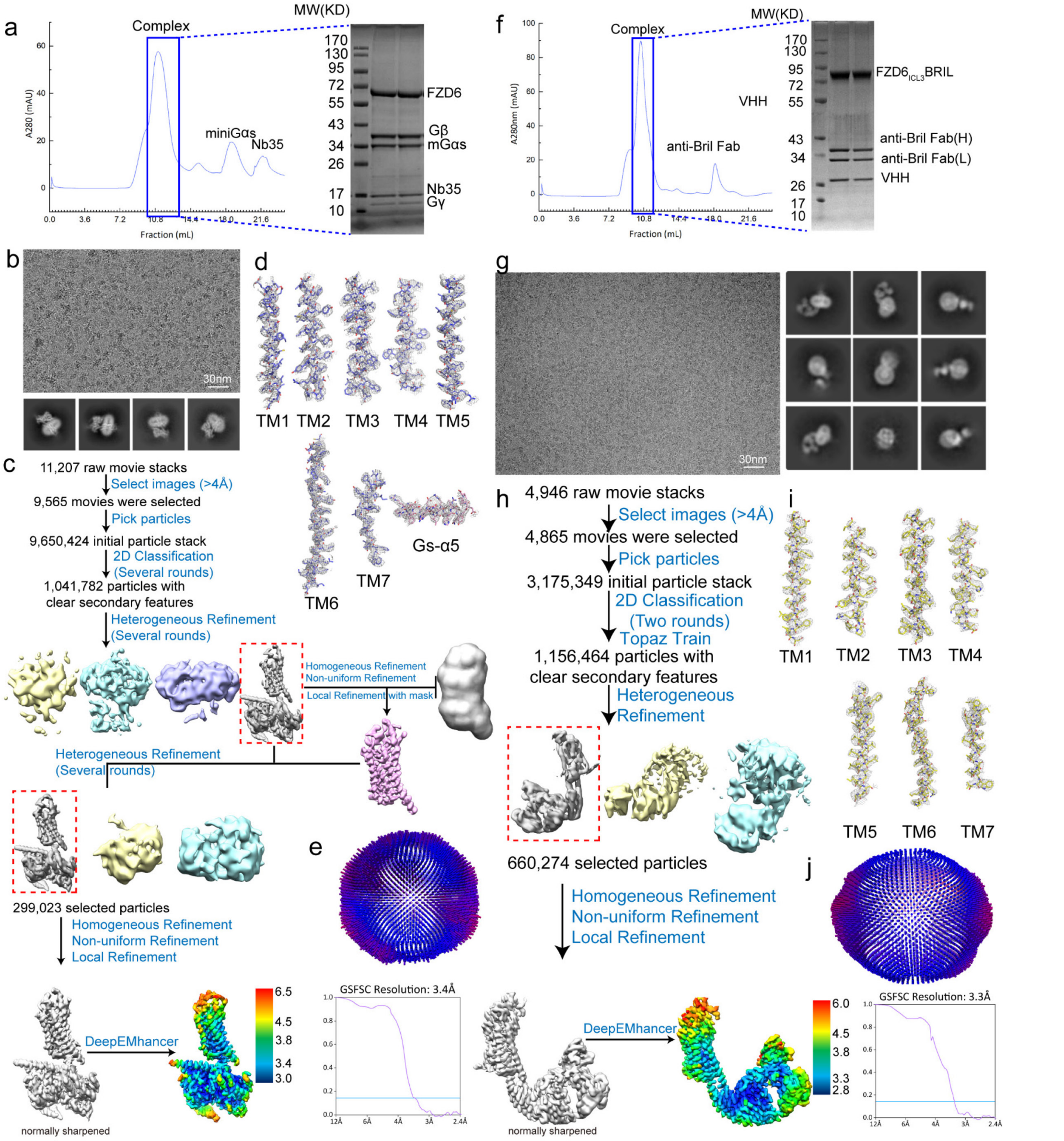

Supplementary Fig. S5 Cryo-EM sample preparation and data processing for FZD6 complexes. a, **f**, Elution profile and gel image of FZD6-Gs (**a**) and inactive FZD6 (**f**) complexes. **b**, **g**, Representative cryo-EM image and representative 2D averages of the FZD6-Gs (**b**) and inactive FZD6 (**g**) complexes**. c**, **h**, Cryo-EM data processing workflow of FZD6-Gs (**c**) and inactive FZD6 (**h**). The data was processed by CryoSPARC and final 3D density maps are colored according to the local resolution. Gold-standard FSC curves from CryoSPARC indicate overall nominal resolutions of 3.40 Å and 3.30 Å using the FSC = 0.143 criterion for the FZD6-Gs (**c**) and inactive FZD6 (**h**) structures. **d**, **i**, Cryo-EM density maps and models are shown for all transmembrane helices and α5 in the Gαs protein for FZD6-Gs (**d**) and inactive FZD6 (**i**) structures, respectively. **e, j**, Angular distribution of the particles used for the final reconstruction of the FZD6-Gs complex (**e**) and inactive FZD6 (**j**) complex.

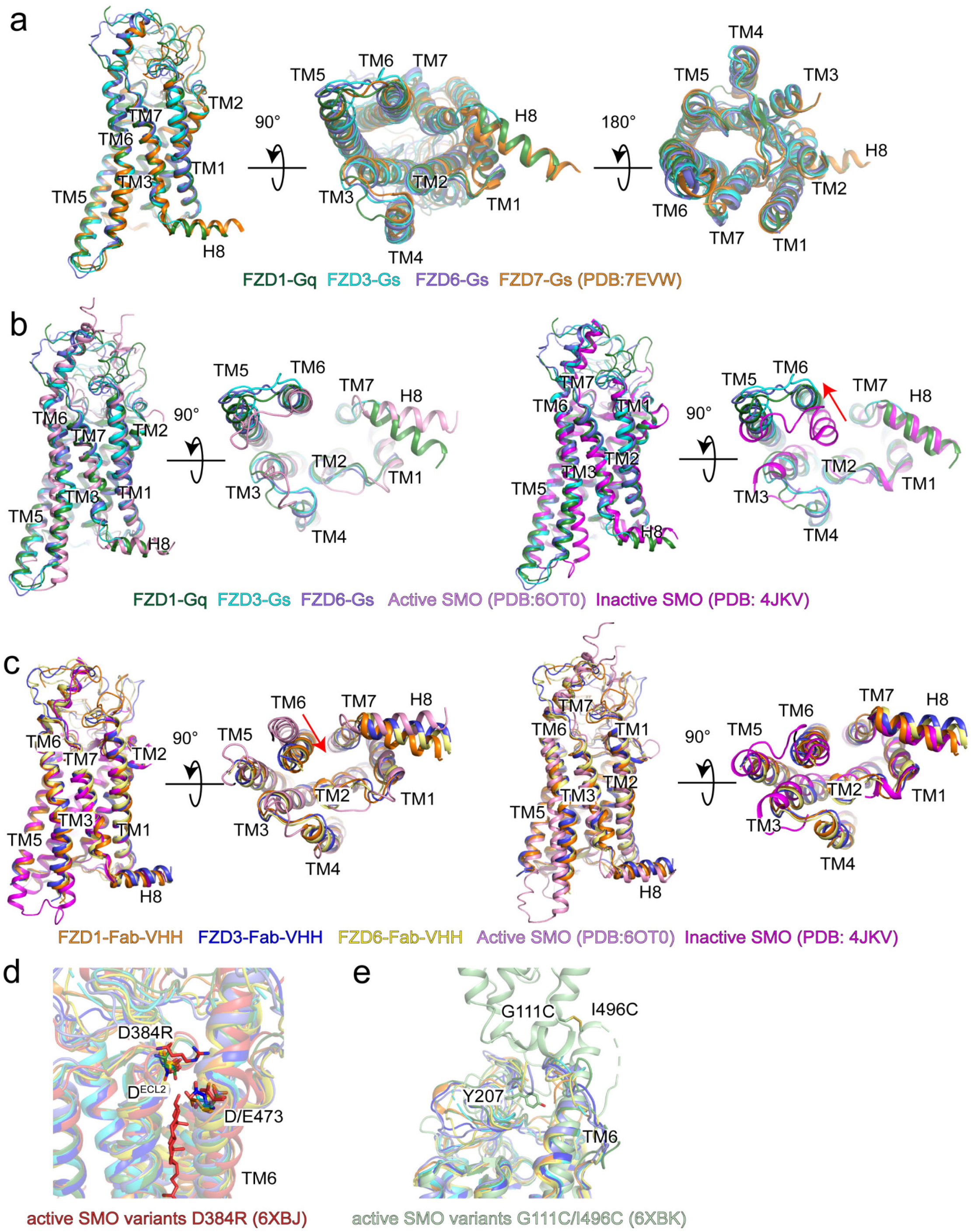

Supplementary Fig. S6 Structural comparison of FZD1-Gq, FZD3-Gs, FZD6-Gs complexes with active and inactive smoothened receptor (SMO) structures. a, Side (left), intracellular (middle) and extracellular (right) views of the overlay between FZD1-Gq, FZD3-Gs, FZD6-Gs and FZD7-Gs structures. **b,** Side and intracellular views of FZD1-Gq, FZD3-Gs, FZD6-Gs with active (left, pink) and inactive (right, magenta) SMO. **c,** Side and intracellular views of FZD1-Fab-VHH, FZD3-Fab-VHH, FZD6-Fab-VHH with inactive (left, pink) and active (right, magenta) SMO. Transmembrane helices TM1-TM7 and helix 8 (H8) are labelled. **d-e,** Structural comparison of Hinge

Domain (HD) and TM6 in FZD1-Gq, FZD3-Gs, FZD6-Gs, FZD1-Fab-VHH, FZD3-Fab-VHH, FZD6-Fab-VHH with constitutively active SMO variants D384R (**d**, red) and G111C/I496C (**e**, palegreen). Residue Y207 in SMO and residues in the position of SMO D384R and D473 are represented as sticks. Color coding is annotated for each protein component.

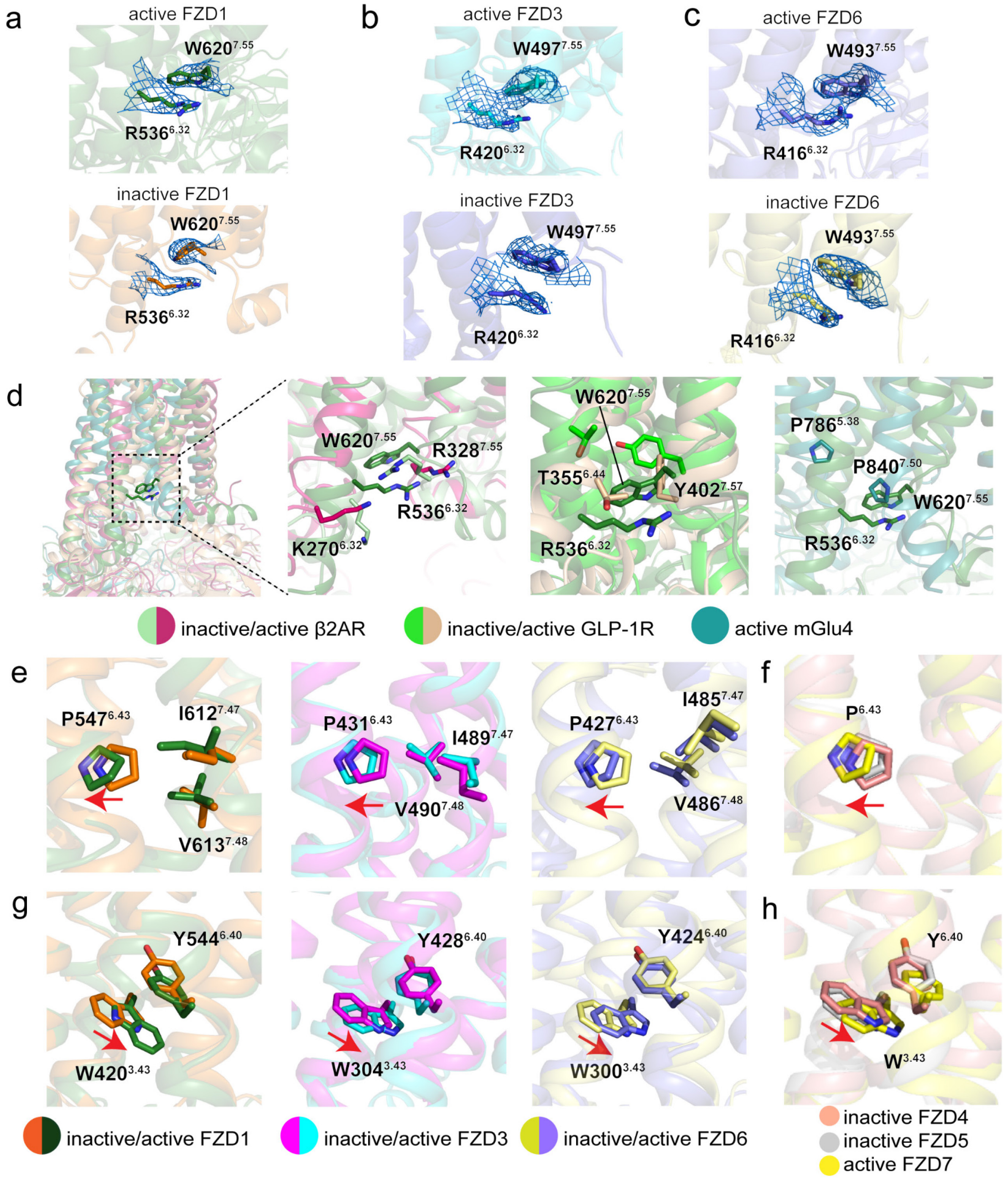

i

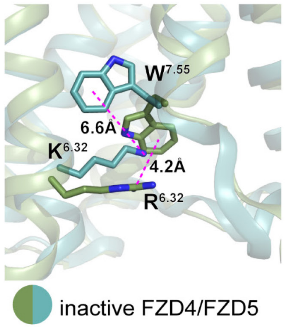

j

	6.32	7.55	6.43	7.47	7.48	3.43	6.40
FZD3	R	W	P	I	V	W	Y
FZD6	R	W	P	I	V	W	Y
FZD5	R	W	P	V	V	W	Y
FZD8	R	W	P	V	V	W	Y
FZD1	R	W	P	I	V	W	Y
FZD2	R	W	P	I	V	W	Y
FZD7	R	W	P	I	V	W	Y
FZD4	K	W	P	L	V	W	Y
FZD9	K	W	P	V	V	W	Y
FZD10	R	W	P	V	V	W	Y
SMO	R	W	F	G	T	F	A

Supplementary Fig. S7 Activation motifs of Frizzled receptors. a-c, The side chains of R$^{6.32}$-W$^{7.55}$ motif in the active (above) and inactive (below) FZD1 (**a**), FZD3 (**b**) and FZD6 (**c**) structures are shown as sticks and overlaid with electron densities (blue mesh).

d, comparison between the structure of FZD1 and other classes of receptors (from left to right, β2AR, GLP-1R, mGlu4) with a focus on comparison of the overlaid positions in accordance with the class-F molecular switch W7.55 and R6.32 (residues shown in dark green) in these three pairs of structures (inactive/active β2AR PDB: 5X7D/7BZ2); inactive/active GLP-1R PDB: 5VEW/5VAI; active mGlu4 PDB: 7E9H). **e,** The conformational rearrangement of residues in kink $P^{6.43}$ of FZD1 (left), FZD3 (middle) and FZD6 (right) upon G protein coupling. **f,** The conformation of residue $P^{6.43}$ in inactive FZD4 (PDB: 6BD4), inactive FZD5 (PDB: 6WW2) and active FZD7 (PDB: 7EVW) structures. **g,** The conformational rearrangement of residues in $W^{3.43}$-$Y^{6.40}$ motif of FZD1 (left), FZD3 (middle) and FZD6 (right) upon G protein coupling. **h,** The conformation of $W^{3.43}$-$Y^{6.40}$ motif in inactive FZD4, inactive FZD5 and active FZD7 structures. **i,** The conformational comparison of R/$K^{6.32}$-$W^{7.55}$ pairs in FZD4 and FZD5. **j,** Sequence alignment of $R^{6.32}$-$W^{7.55}$, kink $P^{6.43}$ and $W^{3.43}$-$Y^{6.40}$ in all ten FZDs and SMO.

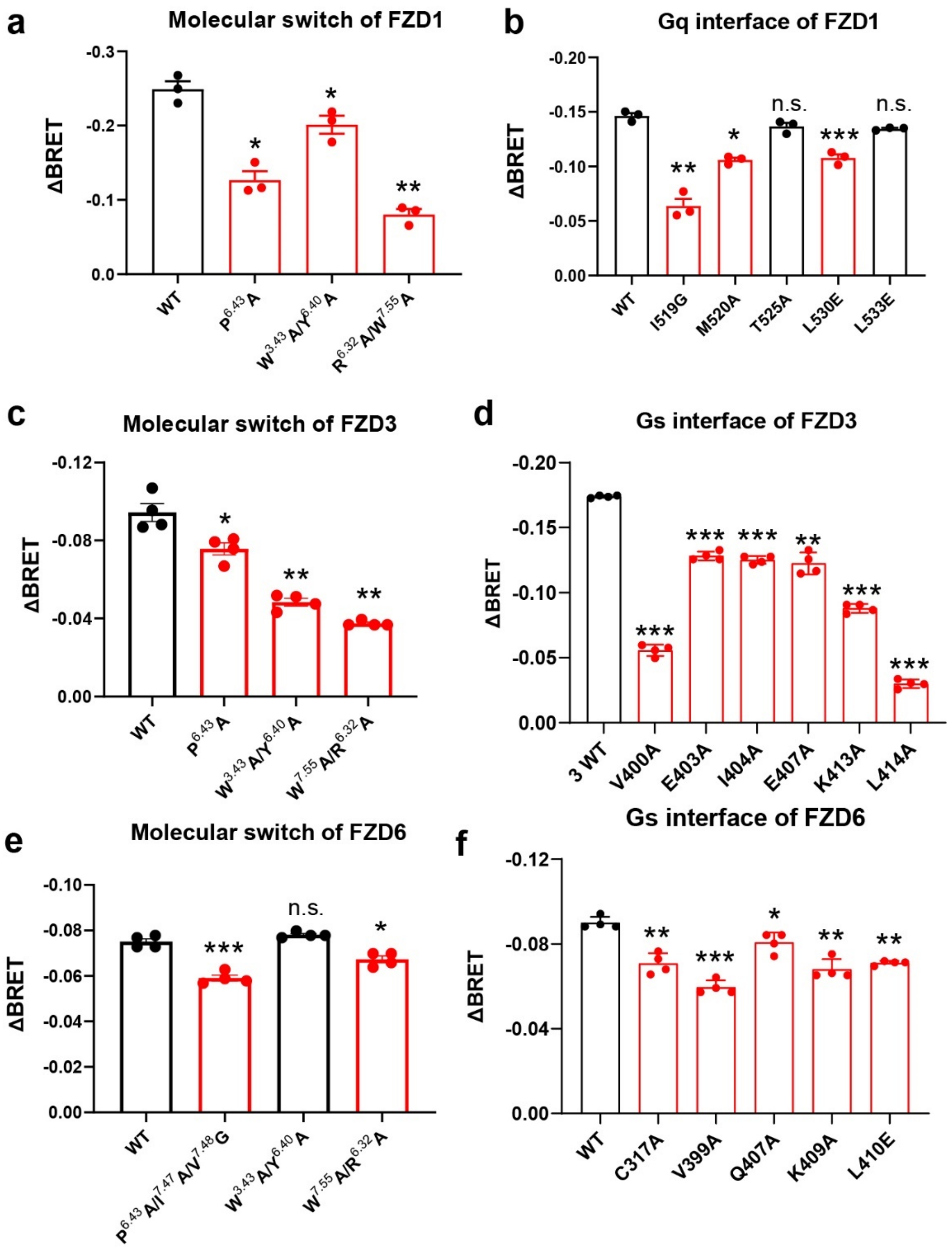

Supplementary Fig. S8 BRET assay of FZD mutants. a, **b**, The effects of activation motifs (**a**) and Gq interface (**b**) mutations on FZD1's constitutive activity are measured by BRET assay. **c**, **d**, The effects of activation motifs (**c**) and Gs interface (**d**) mutations on FZD3's constitutive activity are measured by BRET assay. **e**, **f**, The effects of activation motifs (**e**) and Gs interface (**f**) mutations on FZD6's constitutive activity are measured by BRET assay. Significance was determined by two way ANOVA with Two-stage Benjamini, Krieger, & Yekutieli FDR procedure (***P < 0.001, **P < 0.01, *P < 0.05, n.s. (not significant)). Data are mean ± s.e.m. (n≥3 biologically independent experiments).

	12.51	ICL2	4.35	5.72	5.75	5.76	ICL3	ICL3	6.23	6.25	6.26	6.29
FZD3	R	W	S	V	E	I	E	E	Q	K	L	F
FZD6	R	W	C	V	V	I	D	R	Q	K	L	F
FZD1	S	W	H	I	I	M	D	T	T	K	L	L
FZD2	R	W	H	I	I	M	D	T	T	K	L	L
FZD7	S	W	H	I	I	M	D	T	T	K	L	L
FZD5	R	W	N	I	V	I	G	T	T	K	L	L
FZD8	K	W	N	I	V	I	G	T	T	K	L	L
FZD4	S	W	H	I	N	L	D	T	T	K	L	L
FZD9	Q	W	H	I	I	M	G	T	T	K	L	L
FZD10	R	W	H	I	V	M	G	E	T	K	L	L
SMO	R	Y	·	I	N	H	S	K	A	K	I	T

Supplementary Fig. S9 Sequence alignment of the ten FZDs and SMO at the G protein interface region. Colors represent the properties of residues: blue background: basic; pink background: acidic; green background: polar; yellow background: nonpolar.

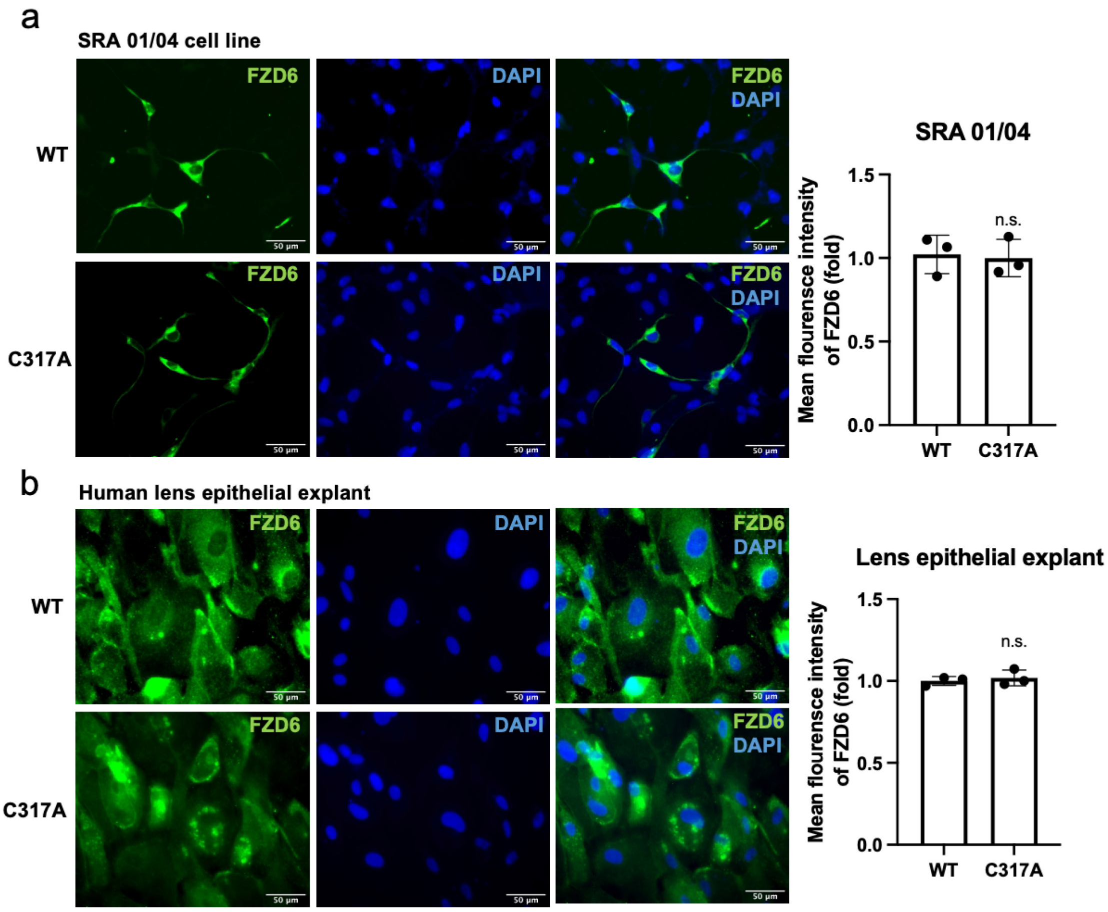

Supplementary Fig. S10 Surface expression of FZD6 in lens epithelial cells. a, Immunofluorescent staining of FZD6 (green) of the human lens epithelial cell line SRA 01/04 transfected with WT FZD6 or the indicated FZD6 mutant. **b,** Immunofluorescent staining of FZD6 (green) of the an primary cultured cells of the human lens epithelial explants treated with 200ng/mL FGF-2 to introduce fiber differentiation and then transfected with WT FZD6 or the indicated FZD6 mutant. The nuclear counter stain is DAPI. Scale bar: 50 μm. Data are representative images of three independent experiments. Mean flourensce intensity was measured by imageJ. Data are mean ± s.e.m. (n = 3). Significance was determined by unpaired Student's t test. n.s. = not significant.

Supplementary Table S1. Cryo-EM data collection, refinement and validation statistics.

	FZD1-Gq	FZD3-Gs	FZD6-Gs	FZD1-Fab-VHH	FZD3-Fab-VHH	FZD6-Fab-VHH
**Data collection and processing**						
Magnification	29,000	105,000	105,000	105,000	105,000	105,000
Voltage (kV)	300	300	300	300	300	300
Electron exposure (e^-/ Å^2)	60	60	60	60	60	60
Defocus range (μm)	-0.7 to -2.2	-0.7 to -2.2	-0.7 to -2.2	-0.7 to -2.2	-0.7 to -2.2	-0.7 to -2.2
Pixel Size (Å)	1.06	0.832	0.832	0.832	0.832	0.832
Symmetry imposed	C1	C1	C1	C1	C1	C1
Initial particle images (no.)	3,453,712	2,968,641	9,650,424	2,573,605	3,350,500	3,175,349
Final particle images (no.)	544,329	89,795	299,023	628,701	221,700	660,274
Map resolution (Å)	3.6	3.5	3.4	3.5	3.4	3.3
FSC threshold	0.143	0.143	0.143	0.143	0.143	0.143
Map resolution range (Å)	3.0 ~ 7.0	3.0 ~ 7.0	3.0 ~ 5.5	2.8~6.5	2.8 ~ 6.5	2.8-6.5
**Refinement**						
Map sharpening B factor (Å^2)	-121	-94.6	-133.1	-142.6	-121.9	-81.9
Model composition						
Non-hydrogen atoms	8483	8562	8688	7441	7563	7663
Protein residues	1107	1077	1098	999	986	988
Ligands	0	0	0	0	0	0
B factors (Å^2)						
protein	69.28	100.53	78.02	88.73	81.77	73.14
Ligand	N/a	N/a	N/a	N/a	N/a	N/a
R.m.s. deviations						
Bond lengths (Å)	0.004	0.004	0.003	0.003	0.004	0.003
Bond angles (°)	0.869	0.704	0.747	0.748	0.714	0.771
Validation						
MolProbity score	1.78	1.75	1.68	1.78	1.68	1.69
Clash score	6.75	6.98	6.37	7.19	5.36	6.27
Poor rotamers (%)	0.00	0.00	0.00	0.00	0.00	0.00
Ramachandran plot						
Favored (%)	93.96	94.60	95.28	94.40	94.24	94.97
Allowed (%)	6.04	5.40	4.72	5.60	5.76	5.03
Disallowed (%)	0.00	0.00	0.00	0.00	0.00	0.00

Supplementary Table S2. Constitutive activity of FZD mutants, measured by BRET assays.

		Constructs	G-protein dissociation (ΔBRET)	P value	Expression	P value
	Activation	WT FZD1	-0.2492±0.0152		100.0±8.8	
	motifs	$P^{6.43}A$	-0.1266±0.0171*	0.0152	92.5±10.33	0.5286
		$W^{3.43}A/Y^{6.40}A$	-0.2011±0.0172*	0.0327	81.7±3.6	0.0723
		$R^{6.32}A/W^{7.55}A$	-0.0802±0.0104**	0.0015	85.9±19.6	0.2338
	Gq	WT FZD1	-0.1464±0.0038			0.2601
FZD1	interface	I519G	-0.0637±0.0095**	0.0056	104.0±7.3*	0.0462
		M520A	-0.1061±0.0029*	0.0106	80.0±2.6	0.1101
		T525A	$-0.1367±0.0048^{n.s.}$	0.1974	85.1±16.1	0.9388
		L530E	-0.1079±0.0048***	0.0003	99.7±9.6	0.3565
		L533E	$-0.1346±0.0010^{n.s.}$	0.0611	94.7±13.0	0.5286
	Activation	WT FZD3	-0.0943±0.0079			
	motifs	$P^{6.43}A$	-0.0757±0.0054*	0.0145	100±8.9	0.2158
		$W^{3.43}A/Y^{6.40}A$	-0.0484±0.0034**	0.0060	139.5±26.1	0.5794
		$R^{6.32}A/W^{7.55}A$	-0.0375±0.0012**	0.0014	111.7±14.6	0.2759
	Gs	WT FZD3	-0.1740±0.0008			
FZD3	interface	K413A	-0.0880±0.0029***	P<0.0001	83.9±6.1	0.1120
		E407A	-0.1225±0.0072**	0.0014	143.5±13.3	0.1771
		E403A	-0.1283±0.0029***	0.0001	72.2±7.4	0.9719
		V400A	-0.0558±0.0037***	P<0.0001	100.4±6.8	0.4581
		I404A	-0.1253±0.0026***	P<0.0001	109.0±16.5	0.1362
		L414A	-0.0300±0.0029***	P<0.0001	57.4±4.72[a]	0.0677
	Activation	WT FZD6	-0.0750±0.0023		100±8.9	
	motifs	$P^{6.43}A/I^{7.47}A/V^{7.48}G$	-0.0590±0.0023***	0.0007	115.1±3.2	0.1641
		$W^{3.43}A/Y^{6.40}A$	$-0.0780±0.0011^{n.s.}$	0.0500	116.8±14.4	0.3344
		$R^{6.32}A/W^{7.55}A$	-0.0673±0.0026*	0.0198	123.2±26.1	0.3692
FZD6	Gs	WT FZD6	-0.0900±0.0025			
	interface	K409A	-0.0680±0.0042**	0.0070	102.2±8.4	0.8450
		Q407A	-0.0808±0.0041*	0.0205	80.0±6.1	0.1344
		V399A	-0.0595±0.0029***	0.0009	76.8±9.5	0.1480
		L410E	-0.0710±0.0009**	0.0010	94.1±3.2	0.2123
		C317A	-0.0708±0.0042**	0.0016	95.1±6.6	0.6406

Data are mean ± s.e.m. from at least three independent experiments. ***P < 0.001, **P < 0.01, *P < 0.05, n.s. (not significant) by two-way ANOVA with Two-stage Benjamini, Krieger, & Yekutieli FDR procedure compared to the response of wild type. The expression levels of the majority of mutants are comparable to that of WT receptor (ranging from 70% to 145% of the WT expression level).

[a]The expression level of the FZD3 mutant L414A is relatively low, and this factor should be considered when discussing the observed reduction in constitutive activity.

Supplementary Table S3. PCP assays of FZD6 mutants.

	Constructs	TOPFlash	P value	ATF2	P value
WT	WT FZD6	1.0000±0.1350		1.0000±0.1750	
Control	FZD6 shRNA	0.7568±0.0407**	0.0083	0.3680±0.0221****	<0.0001
Activation motifs	$R^{6.32}A/W^{7.55}A$	0.9121±0.0545 $^{n.s.}$	0.5093	0.6504±0.0434**	0.0023
Gs interface	C317	0.8545±0.0180 $^{n.s.}$	0.1303	0.7560±0.0050*	0.0265
	Q407A	0.8936±0.0993 $^{n.s.}$	0.345	0.4097±0.0407****	<0.0001

	Constructs	mRNA of FZD6	P value	mRNA of RHOA	P value
WT	WT FZD6	1.0040±0.1119		1.0003±0.0292	
Control	FZD6 shRNA	0.4312±0.0335****	<0.0001	0.6245±0.1176***	0.0008
Activation motifs	$R^{6.32}A/W^{7.55}A$	0.8731±0.0765 $^{n.s.}$	0.5046	0.7527±0.0319*	0.034
Gs interface	C317	0.8088±0.0721 $^{n.s.}$	0.1347	0.7622±0.0621*	0.0442
	Q407A	0.7708±0.0812 $^{n.s.}$	0.0505	0.7545±0.0481*	0.0357

Data are mean ± s.e.m. from at least three independent experiments. ****P < 0.0001, ***P < 0.001, **P < 0.01, *P < 0.05, n.s. (not significant) by two-way ANOVA with Two-stage Benjamini, Krieger, & Yekutieli FDR procedure compared to the response of wild type.
